# Optimizing Diabetes Diagnosis Through Pulse Waveform Analysis and Data Mining

**DOI:** 10.3390/bioengineering12121287

**Published:** 2025-11-23

**Authors:** Shun-Chang Chang, Ruei-Yu Lin, Shiaw-Meng Chang, Li-Chun Teng, Tien-Hsiung Ku, Wei-Chang Yeh, Chia-Ling Huang

**Affiliations:** 1 Department of Chinese Medicine, Changhua Christian Hospital, Changhua 500, Taiwan; 140816@cch.org.tw (S.-C.C.); marknspsbl@gmail.com (S.-M.C.); 131379@cch.org.tw (L.-C.T.); 2 Department of Industrial Engineering and Engineering Management, National Tsing Hua University, Hsinchu 300, Taiwan; caroline0921@gapp.nthu.edu.tw; 3 Changhua Christian Hospital, Changhua 500, Taiwan; 21203@cch.org.tw; 4 Department of Industrial and Systems Engineering, College of Electrical Engineering and Computer Science, Chung Yuan Christian University, Taoyuan 320, Taiwan; 5 Department of International Logistics and Transportation Management, Kainan University, Taoyuan 338, Taiwan; clhuang@mail.knu.edu.tw

**Keywords:** pulse wave, traditional Chinese medicine, tree algorithm, Stacking ensemble learning

## Abstract

The objective of this study is to develop a robust diabetes diagnosis model by employing four distinct algorithms as weak learners: the Random Forest algorithm, SVM algorithm, KNN algorithm, and Decision Tree algorithm. The selection of the optimal classification model involves a meticulous process, and further refinement is conducted through the application of the Stacking classifier, with the Multilayer Perceptron (MLP) classifier serving as the final model. The performance of the optimized model is thoroughly evaluated to identify the most effective diagnostic model. The experiments are conducted using a dataset obtained from Changhua Christian Hospital in Taiwan. Our experimental results show that the performance of the model optimized by the Stacking ensemble learning method is significantly improved. The optimized model achieves an *F*_1_ score of 0.86 and an AUC score of 0.90, indicating the effectiveness of the proposed model.

## 1. Introduction

Traditional Chinese Medicine (TCM), known for its rich experience and theories, is widely practiced around the world. Based on holistic concepts of the body and spirit, overall assessment, and the four diagnostic methods, TCM has developed into a unique and effective system of treatment [1].

TCM physicians employ four diagnostic approaches—inspection, auscultation and olfaction, inquiry, and palpation—to evaluate the health status of individuals. These time-honored methods rely heavily on sensory systems, incorporating vision, olfaction, audition, and touch, all guided by the principles of TCM theory. These diagnostic techniques, deeply intertwined with TCM’s holistic philosophy, continue to be actively utilized in contemporary practice [2]. However, the subjectivity inherent in these methods, influenced by the individual capabilities and experiences of TCM physicians, may result in diagnostic variations among different physicians [3].

Since the 1980s, Traditional Chinese Medicine (TCM) has shifted from relying only on traditional diagnostic methods to exploring pulse diagnosis waveforms. Modern studies have found that reduced arterial elasticity and poor endothelial function are closely linked to diabetes. Because diabetes weakens blood vessel flexibility and raises blood pressure, pulse waveforms can be used to assess a patient’s health [4]. This shift has led to the development of advanced instruments, the creation of pulse diagnosis rooms in major TCM hospitals, and the modernization of pulse diagnosis practices. As TCM continues to modernize and gain international attention, its value is increasingly recognized worldwide. Modern intelligent technologies are now being used to standardize and digitize TCM diagnostic and treatment methods [5].

Data mining methods are used to search for patterns and trends in large-scale datasets, facilitating data partitioning and the evaluation of the probability of future events. Data Mining encompasses various disciplines, including statistics, probability, artificial intelligence (AI) [1]. The application of AI in the medical field has become widespread, leveraging its real-time and precise characteristics to mitigate risks, enhance efficiency, significantly improve healthcare quality, and provide opportunities for medical advancement [6]. The healthcare industry continuously generates and stores vast amounts of new data [2]. Effectively harnessing this data can assist professionals in delivering rapid and accurate diagnostics [7]. Therefore, in the past, data mining techniques have been used to analyze the performance of different classification algorithms in the early diagnosis of diabetes, and experiments have been conducted using various machine learning methods for early diabetes diagnosis [8,9]. Alarmingly, one in two people live with diabetes without awareness of their condition. Unfortunately, their health status remains unmonitored and uncontrolled. According to research statistics, in 2019, an estimated 463 million people worldwide had diabetes. The prevalence of diabetes is projected to reach 578 million by 2030 and 700 million by 2045 [10]. Additionally, people with diabetes are more likely to experience severe symptoms and complications when infected with COVID-19 [11].

This study integrates ten years of TCM pulse diagnosis data and applies Data Mining techniques using four algorithms—Random Forest, SVM, KNN, and Decision Tree—as base models. The aim is to build a strong diabetes diagnosis model that improves the accuracy of modern TCM pulse diagnosis instruments. The expected outcome is to provide a useful tool for more accurate diabetes detection, which may help save lives in the future. Overall, the results show that the Random Forest algorithm performs better than the others in learning, prediction, and classification. In addition, the model further optimized using the Stacking ensemble method shows significantly improved performance compared with the Random Forest algorithm alone.

By combining four distinct weak learners—Random Forest, SVM, KNN, and Decision Tree—with a Multilayer Perceptron as the meta-classifier, the study introduces a robust and data-driven approach that enhances diagnostic accuracy and model generalization. The use of real-world medical data from Changhua Christian Hospital further strengthens the practical relevance and applicability of the proposed method. The experimental results, with an *F*_1_ score of 0.86 and an AUC of 0.90, highlight a significant improvement over individual base models, underscoring the effectiveness of ensemble optimization in medical prediction tasks. Therefore, the results lie in the field of the feature selection and experimental proof that the considered data enable us to predict the diabetes.

The other sections are as follows: Section 2 is Optimal Model Design. The Experimental Process is presented in Section 3. Experimental Evaluation and Analysis are in Section 4. Section 5 is Conclusions.

## 2. Optimal Model Design

**Figure 1:**
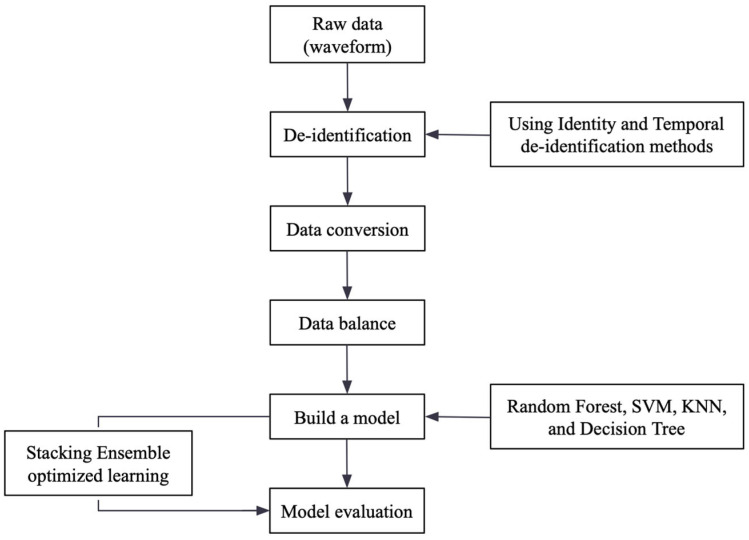
Optimized model design framework.

Developing an accurate diabetes diagnosis model requires effective data preprocessing and model optimization. Figure 1 shows how these two processes work together in the model framework.

The diabetes diagnosis model framework encompasses four critical steps to ensure a comprehensive and effective approach.

Step 1 Initially, data preprocessing is performed to clean, transform, and normalize the raw data from text files, ensuring its integrity and preparing it for subsequent modeling. This step involves extracting key statistical and frequency domain features that capture crucial characteristics of pulse waveforms.

### 2.1. Data Cleaning

To keep a consistent link between pulse waveforms and HbA1c values, data cleaning was done during preprocessing. The time gap between the waveform and HbA1c tests was checked, since a long gap could weaken their correlation and reduce model accuracy. Records with intervals over 200 days were removed to ensure reliable data.

To improve data quality, each pulse waveform needed at least three full pulse cycles. Records with fewer cycles were removed because they were more affected by noise and could distort feature calculations. After cleaning, data with poor timing or unstable waveforms were excluded, resulting in a more consistent dataset suitable for normalization and transformation, and improving model accuracy and stability.

### 2.2. Normalization

To reduce the effect of feature scale differences on model training, all numerical features were Z-score normalized, converting them to a standard normal distribution with mean 0 and standard deviation 1 (Equation (1)).

(1) $$z=\frac{x-u}{\sigma }$$

In this equation, *x* represents the original feature value, while *μ* and *σ* denote the mean and standard deviation of the corresponding feature in the training set, respectively. This process ensures that all features share a common scale, preventing features with larger magnitudes from dominating the model during training. Moreover, the *μ* and *σ* values computed during the training phase were also applied to the test set to maintain consistency in data preprocessing and ensure fairness in model evaluation.

### 2.3. Logarithmic Transformation

Table 1 presents the summary statistics of the original distribution of the hospital’s glycated hemoglobin (HbA1c) dataset used in this study.

The data were mostly between 6 and 8, with a right-skewed distribution and some outliers. To reduce the range and lessen outlier effects, *HbA1c* values were log-transformed (Equation (2)).

*y* = log (*HbA*1*c* + 1) (2)

In this equation, *y* denotes the transformed indicator, and *HbA*1*c* represents the original blood glucose measure. Adding 1 before applying the logarithm prevents domain issues for values close to zero. After the logarithmic transformation, the sample distribution becomes more symmetrical, with a significant reduction in overall skewness. This transformation facilitates faster convergence and improved predictive performance of machine learning models, while also enhancing the model’s robustness to outliers.

Step 2 After preprocessing, feature selection identified the most relevant features for diabetes classification, including mean, variance, peaks, max height, peak angle, first valley percentage, spectral energy, and max PSD frequency.
Step 3 The selected features are then input into an ensemble of diverse algorithms, including the Random Forest algorithm, Support Vector Machine (SVM) algorithm, k-Nearest Neighbors (KNN) algorithm, and Decision Tree algorithm. Random Forest is a classifier that operates by constructing a collection of decision trees, where each tree is built using a different random subset of the data. In this model, the input variable XX serves as the basis for each individual tree, allowing the model to capture a diverse range of patterns in the data [12]. SVMs are a class of supervised learning techniques used to detect, identify, or regress shapes [13]. The KNN method can automatically determine the optimal value of k, reducing dependency on k and enhancing classification efficiency while maintaining high accuracy [14]. The Decision Tree is a widely utilized classification model. As a statistical tool for data processing, DT operates by utilizing a tree-like structure to make decisions [15]. This ensemble learning approach leverages the strengths of each algorithm to create a robust and versatile diabetes diagnosis model.
Step 4 The model is trained on the preprocessed data and evaluated on a separate test set for diabetes prediction. Stacking was used for the ensemble because it combines multiple base models through a meta-learner, improving generalization, stability, and reducing bias or variance. This framework integrates preprocessing, feature selection, and ensemble learning to deliver accurate and reliable predictions.

## 3. Experimental Process

### 3.1. Data Set Analysis and Preprocessing

The dataset includes 2938 cases: 1277 with diabetes and 1661 without, covering diabetic, non-diabetic, and other medical conditions. It has 8 pulse waveform-related features and a class attribute indicating whether each subject has diabetes.

The data were collected from patients at the TCM Clinic of Changhua Christian Hospital (2010–2020) using the ANSWatch device on the left radial artery. Pulse waveforms and parameters—including blood pressure, heart rate, HRV, LF%, HF%, LF/HF, and irregular heartbeat count over 5 min—were recorded. Table 2 lists the dataset attributes and their meanings.

To improve data quality, noisy instances were removed, and features were standardized to follow a normal distribution. Since there were no missing values, data imputation was not needed.

To address class imbalance, minority class (diabetes) instances were oversampled to match the majority class. This helps reduce bias and improves the model’s ability to detect diabetes cases.

### 3.2. Experimental Setup

After preprocessing, the dataset was split into training (80%) and test sets (20%). This ensures a comprehensive evaluation of the model’s performance. The training set allows the model to learn data intricacies, while the test set assesses its performance on unseen data.

The study includes two main experiments. The first compares four classifiers—Random Forest, SVM, KNN, and Decision Tree—to find the best performer. Models are trained on a training set and tested for accuracy, with the top results later compared to the stacked model.

In the next experiment, Random Forest, SVM, KNN, and Decision Tree serve as weak learners to build a stacked ensemble model. Their predictions are combined by a final estimator, improving stability and accuracy on new data.

## 4. Experimental Evaluation

### 4.1. Feature Importance Analysis

**Figure 2:**
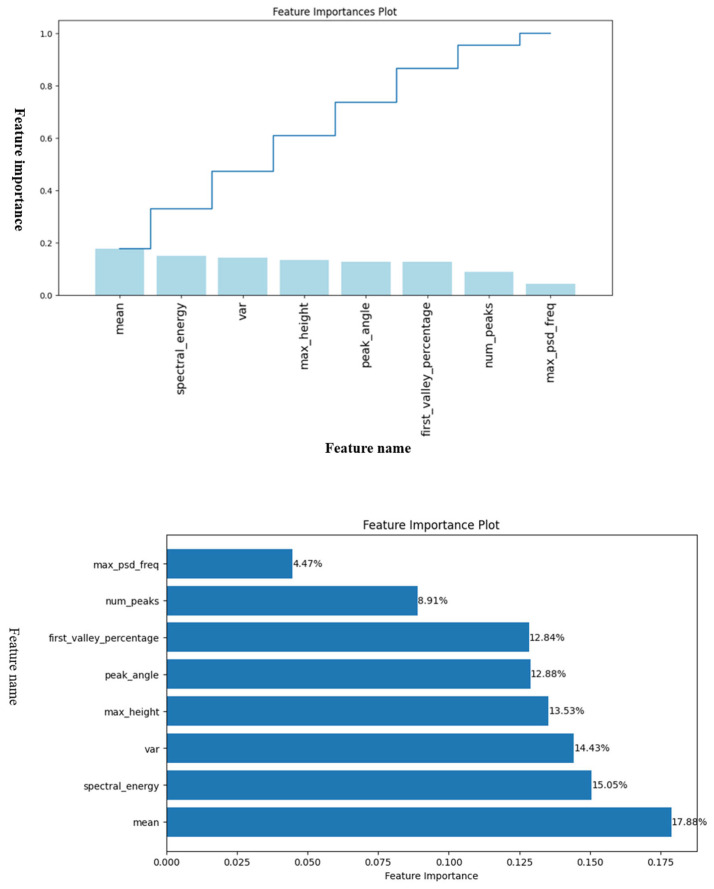
Feature importance plot.

Feature importance analysis evaluates how much each feature contributes to predicting the target. Figure 2 shows the Random Forest’s feature importance, highlighting which features most influence predictions and improving model credibility.

In this study, we used all 8 features. Table 3 presents the feature ranking according to Importance Ratio. The three most relevant features for this dataset are mean value of the pulse waveform, spectral energy, and variance of the pulse waveform.

### 4.2. Performance Metrics

Performance metrics provide standardized ways to evaluate a model’s effectiveness. This study uses AUC, accuracy, Precision, Recall/Sensitivity, Specificity, and *F*_1_-Score to assess performance from multiple angles. AUC measures class discrimination, accuracy shows overall correctness, Precision and Recall balance false positives and negatives, Specificity reflects correct identification of negatives, and *F*_1_-Score combines Precision and Recall for imbalanced data. Together, these metrics ensure a thorough and clinically reliable evaluation.

The Confusion Matrix correlates the true class of all the samples to be predicted with the predicted class. In the binary classification problem, we can divide the sample into four categories according to the confusion matrix. This is shown in the following Table 4.

AUC is the area under the ROC (Receiver Operating Characteristic) curve, ranging from 0 to 1 [16]. It measures the model’s ability to correctly classify true positives and true negatives at different thresholds. A higher AUC value indicates better performance in predicting true positives and true negatives. ROC is an important metric for evaluating classification model performance. It is a graphical representation with the true positive rate on the vertical axis and the false positive rate on the horizontal axis. The ROC curve provides insights into the trade-off between sensitivity and specificity.

Accuracy represents the proportion of samples that the model correctly predicted compared to the total number of samples.

(3) $$Accuracy=\frac{TP+TN}{TP+TN+FP+FN}$$

Precision measures how many of the predicted positive instances are truly positive. It is defined as the number of true positives divided by the number of true positives plus the false positives.

(4) $$Precision=\frac{TP}{TP+FP}$$

Recall, also known as Sensitivity or True Positive Rate, measures the proportion of actual positive instances that the model correctly identifies. It is defined as the number of true positives divided by the number of true positives plus the number of false negatives.

(5) $$Sensitivity=\frac{TP}{TP+FN}$$

Specificity measures the ability of a classification model to correctly identify negative instances or the true negative rate. Specificity defines the true negative rate, which is the proportion of actual negative instances that were correctly predicted. To calculate this metric, we divide the number of false positives by the sum of true negatives and false positives.

(6) $$Specification=\frac{TN}{TN+FP}$$

*F*_1_ score is the harmonic mean of Precision and Recall, providing a comprehensive assessment that considers the performance of both.

(7) $$F_{1}=2\cdot \frac{precision\cdot sensitivity}{precision+sensitivity}$$

### 4.3. Experiment Results Based on Different Classifier

After five experiments, the experimental results were averaged, and according to the confusion matrix of four models of Random Forest, SVM, KNN, and Decision Tree algorithms and the above formula, the accuracy, precision, recall, specificity, *F*_1_-Score, and AUC value were obtained as shown in Table 5, and the optimal data in the comparison of various indicators are displayed in bold.

The Random Forest algorithm demonstrated exceptional performance, securing the highest *F*_1_ score of 0.81, outperforming the SVM algorithm, which achieved a comparatively lower *F*_1_ score of 0.52. This highlights the Random Forest algorithm’s superior predictive capability over its counterparts. The remaining algorithms exhibited *F*_1_ scores with variations ranging from 0.06 to 0.29 in comparison to the highest *F*_1_ score. All four models attained AUC values surpassing 0.5, with the minimum recorded at 0.55. This indicates that each model excels in effectively distinguishing between the two classes in the diabetes classification task, surpassing random guessing. Nevertheless, further enhancements are necessary to achieve more satisfactory outcomes in diabetes discrimination. These findings underscore the importance of ongoing refinement to optimize the models for diabetes prediction.

Notably, the Random Forest algorithm achieved the highest AUC value of 0.89, and its accuracy also surpassed that of the other three models, reaching 0.82. In contrast, the SVM algorithm had the lowest accuracy at 0.57, differing by 0.25 from the highest accuracy achieved by the Random Forest algorithm. Although the recall of the Random Forest algorithm did not rank the highest, it secured the second position among the four algorithms.

In summary, the results suggest that the Random Forest algorithm exhibits superior learning, prediction, and classification abilities compared to the other algorithms. However, there is room for improvement, and further refinements are necessary to enhance its performance in diabetes prediction.

### 4.4. Experiments Based on Stacking Ensemble Learning

Upon evaluating the outcomes of Experiment 1, it is evident that the performance of the Random Forest algorithm surpasses that of the others. However, in the pursuit of enhancing the precision of model predictions, these four models are employed as distinct base classifiers (Weak Learners). They serve as the foundational layer in the Stacking model, leveraging ensemble learning techniques to capitalize on the strengths of each base classifier, thereby improving the overall model performance. Following numerous experiments, the averaged experimental results were computed. Utilizing the model’s confusion matrix and the formulas above, Table 6 presents the calculated metrics, including accuracy, precision, recall, *F*_1_ score, and specificity.

The evaluation of the Random Forest model’s performance reveals a notable enhancement following the application of Stacking ensemble learning. The specificity witnessed a substantial increase of approximately 15%, with the optimized Random Forest model achieving a specificity value of 0.96. This result signifies a commendable improvement in identifying negative instances. The precision also exhibited a noteworthy improvement of 14%, reaching a precision value of 0.94 for the optimized model, which is considered exemplary. This improvement indicates that the optimized model achieved a higher ratio of true positive predictions among all positive predictions, showcasing enhanced precision in its classification performance. Additionally, metrics such as accuracy, *F*_1_-score, AUC experienced significant augmentation.

In conclusion, the Stacking ensemble learning technique effectively optimized the Random Forest model, resulting in heightened performance and classification accuracy. The findings suggest a substantial improvement in the model’s learning and generalization capabilities. Overall, the Stacking optimized model emerges as the superior performer, attaining the highest classification accuracy.

## 5. Conclusions

This study successfully developed a robust diabetes diagnosis model by integrating four distinct algorithms—Random Forest, SVM, KNN, and Decision Tree—within a Stacking ensemble framework, using a Multilayer Perceptron as the final classifier. The optimized model achieved an *F*_1_ score of 0.86 and an AUC of 0.90, demonstrating the effectiveness of ensemble learning in enhancing predictive performance. Beyond the quantitative improvements, these findings have practical relevance for clinical decision support: by enabling more accurate and early detection of diabetes, the model can assist healthcare providers in identifying at-risk patients, informing timely interventions, and ultimately improving patient outcomes. The integration of such models with clinical workflows could streamline diagnostic processes and complement existing healthcare practices.

Utilizing data mining is vital for enhancing clinical decision support systems, as medical institutions actively amass extensive datasets to support healthcare facilities and public health initiatives. Early disease detection can significantly impact an individual’s long-term quality of life. This study aimed to compare the performance of Random Forest, SVM, KNN, and Decision Tree algorithms, identifying the best-performing models based on *F*_1_ and AUC evaluations in Experiment 1. In Experiment 2, Stacking ensemble learning optimized the model, resulting in significant improvements with *F*_1_ and AUC values of 0.86 and 0.90, respectively. The optimized Stacking model demonstrated superior performance, emphasizing the importance of ensemble learning for enhancing diabetes prediction models. Despite limitations in patient-related information in the dataset, future experiments will incorporate critical features, fostering a comparative analysis for improved study robustness. This research provides valuable insights for clinical medicine and contributes to the advancement of data mining and machine learning.

However, the study is constrained by the limited scope of patient-related features in the dataset. Future research could address these limitations by incorporating additional physiological parameters, such as heart rate variability or photoplethysmography, as well as data from multiple medical centers to enhance model generalizability. Prospective validation studies are also recommended to assess real-world applicability and reliability. By expanding the range of features and validating the model in diverse clinical settings, future work can further advance the utility of data mining and machine learning for robust, clinically relevant diabetes prediction.

The in-depth theoretical discussion and systematic comparison with related work will be addressed in future planned research. Furthermore, future research will also strengthen the methodological innovation, provide more rigorous analysis, and position the work more clearly within the context of existing literature. Future research will further enhance methodological depth by exploring alternative ensemble schemes such as bagging or boosting, or by employing statistical learning techniques (e.g., logistic regression with regularization, discriminant analysis, or Bayesian classifiers) for comparative analysis. The introduction of k-fold cross-validation or a leave-one-out validation protocol would provide stronger evidence of model stability and reliability. Future research will also include plans to provide more information about data acquisition, calibration, and quality control to strengthen the reproducibility of the study. Additionally, incorporating data from other centers or including more clinical parameters, such as age, body mass index, or blood pressure, would enable the development of a more balanced and representative model. Future research might consider applying correlation analysis, stepwise selection, or dimensionality reduction methods (such as principal component analysis) to identify the most informative parameters. Moreover, discussing the physiological relevance of key features—such as how spectral energy or waveform variance reflect vascular stiffness or metabolic status—would enrich the interpretation. The performance measures analysis would be more convincing if accompanied by statistical tests. Confidence intervals for the reported metrics or comparative significance tests between classifiers would add robustness. Including visual representations, such as receiver operating characteristic (ROC) curves or confusion matrices, could help readers grasp performance differences more intuitively, and these could also be planned for future research. In view of the fact that the only possible innovation in this paper lies in using new datasets and a new set of features, future research could focus on comparing different datasets or combinations of datasets and methods. In future research, adding short explanations for key features and including one or two more figures (for example, ROC curves) would make the paper clearer. Also, a simple validation test or comparison with other studies would strengthen the results. 

## Figures and Tables

**Table 1:** Summary statistics of *HbA*1*c* distribution.

Indicator	Value
Mean	7.1
Standard Deviation	1.3
Minimum	4.4
25th Percentile	6.2
50th Percentile	6.8
75th Percentile	7.6
Maximum	15.4

**Table 2:** Dataset description.

Attribute Name	Description in	Type
Mean	The average amplitude of pulse wave heights	Feature
Variance	The variance of pulse wave amplitude	Feature
Number of peaks	The number of pulse beats	Feature
Max height	The maximum amplitude of the pulse waveform	Feature
Peak angle	The angle formed by connecting the highest peak of the pulse waveform with the first trough that occurs after the highest peak	Feature
First valley percentage	The percentage position of the first valley relative to the entire waveform	Feature
spectral energy	The energy within a specific frequency range of the spectrum	Feature
Max PSD frequency	The maximum frequency of Power Spectral Density	Feature
class	No diabetes or with diabetes	Target

**Table 3:** Ranked Attributes.

Rank	Feature	Importance Ratio
1	Mean	0.1788
2	spectral energy	0.1505
3	Variance	0.1443
4	Max height	0.1353
5	Peak angle	0.1288
6	First valley percentage	0.1284
7	Number of peaks	0.0891
8	Max PSD frequency	0.0447

**Table 4:** Confusion matrix.

Predicted Value	Actual Value	
	Positive Example	Counter Example
Positive example	TP	FP
Counter example	FN	TN

**Table 5:** Results of Evaluation Measures based on the four models.

The Name of Model	Accuracy	Precision	Recall	Specificity	*F*_1_-Score	AUC
Random Forest	**0.82**	**0.80**	0.83	**0.82**	**0.81**	**0.89**
SVM	0.57	0.54	0.50	0.64	0.52	0.55
KNN	0.61	0.57	0.68	0.55	0.62	0.64
Decision Tree	0.74	0.68	**0.84**	0.66	0.75	0.74

**Table 6:** The numeric value of each classifier indicator.

The classifier name	Accuracy	Precision	Recall	*F*_1_	Specificity	AUC
Random Forest	0.82	0.80	0.83	0.82	0.81	0.89
Stacking	**0.86**	**0.94**	0.76	**0.83**	**0.96**	**0.90**

## Data Availability

Data Availability Statements are available in Section 3.1.

## References

[1] Liang Y., Huang Y. (2022). Bian Que, the founder of diagnostics of traditional Chinese medicine. J. Tradit. Chin. Med. Sci..

[2] Jiang M., Lu C., Zhang C., Yang J., Tan Y., Lu A., Chan K. (2012). Syndrome differentiation in modern research of traditional Chinese medicine. J. Ethnopharmacol..

[3] Sui J., Zhang L., Yang F. (2022). Data-driven based four examinations in TCM: A survey. Digit. Chin. Med..

[4] Zhang D., Zuo W., Wang P., Zhang D., Zuo W., Wang P. (2018). Generalized feature extraction for wrist pulse analysis: From 1-D time series to 2-D matrix. Computational Pulse Signal Analysis.

[5] Scuteri D., Corasaniti M.T., Tonin P., Nicotera P., Bagetta G. (2021). New trends in pharmacological control of neuropsychiatric symptoms of dementia. Curr. Opin. Pharmacol..

[6] Yeh W.C., Shia W.C., Hsu Y.T., Huang C.H., Lee Y.S. (2025). A Lightweight Breast Cancer Mass Classification Model Utilizing Simplified Swarm Optimization and Knowledge Distillation. Bioengineering.

[7] Jothi N., Rashid N.A., Husain W. (2015). Data Mining in Healthcare—A Review. Procedia Comput. Sci..

[8] Sun J., Reddy C.K. Big data analytics for healthcare. Proceedings of the 19th ACM SIGKDD International conference on Knowledge Discovery and Data Mining.

[9] Yeh W.C., Chu C.L. (2024). Feature selection for data classification in the semiconductor industry by a hybrid of simplified swarm optimization. Electronics.

[10] Chitra R., Seenivasagam V. (2013). Review of Heart Disease Prediction System Using Data Mining and Hybrid Intelligent Techniques. ICTACT J. Soft Comput..

[11] Chaves L., Marques G. (2021). Data mining techniques for early diagnosis of diabetes: A comparative study. Appl. Sci..

[12] Saeedi P., Petersohn I., Salpea P., Malanda B., Karuranga S., Unwin N., Colagiuri S., Guariguata L., A Motala A., Ogurtsova K. (2019). Global and regional diabetes prevalence estimates for 2019 and projections for 2030 and 2045: Results from the International Diabetes Federation Diabetes Atlas. Diabetes Res. Clin. Pract..

[13] American Diabetes Association (2022). Frequently asked questions: COVID-19 and diabetes. American Diabetes Association. https://www.diabetes.org/coronavirus-covid-19/.

[14] Simic V., Torkayesh A.E., Maghsoodi A.I. (2023). Locating a disinfection facility for hazardous healthcare waste in the COVID-19 era: A novel approach based on Fermatean fuzzy ITARA-MARCOS and random forest recursive feature elimination algorithm. Ann. Oper. Res..

[15] Chidambaranathan S., Radhika A., Priya V.V., Mohan S.K., Gireeshan M.G. (2021). Optimal SVM based brain tumor MRI image classification in cloud internet of medical things. Cognitive Internet of Medical Things for Smart Healthcare: Services and Applications.

[16] Guo G., Wang H., Bell D., Bi Y., Greer K. KNN model-based approach in classification. Proceedings of the Move to Meaningful Internet Systems 2003: CoopIS, DOA, and ODBASE: OTM Confederated International Conferences, CoopIS, DOA, and ODBASE 2003.

